# Paeonol for the Treatment of Atherosclerotic Cardiovascular Disease: A Pharmacological and Mechanistic Overview

**DOI:** 10.3389/fcvm.2021.690116

**Published:** 2021-07-21

**Authors:** Min Wu, Zongliang Yu, Xiaoya Li, Xiaonan Zhang, Songzi Wang, Shengjie Yang, Lanqing Hu, Longtao Liu

**Affiliations:** ^1^Guang'an Men Hospital, China Academy of Chinese Medical Sciences, Beijing, China; ^2^National Clinical Research Center for Chinese Medicine Cardiology, Xiyuan Hospital, China Academy of Chinese Medical Sciences, Beijing, China; ^3^Graduate School, Beijing University of Chinese Medicine, Beijing, China

**Keywords:** *Paeonia suffruticosa*, paeonol, traditional Chinese medicine, atherosclerotic cardiovascular disease, review, pharmacology, mechanism

## Abstract

With improvement in living standards and average life expectancy, atherosclerotic cardiovascular disease incidences and mortality have been increasing annually. *Paeonia suffruticosa*, a natural herb, has been used for the treatment of atherosclerotic cardiovascular disease for thousands of years in Eastern countries. Paeonol is an active ingredient extracted from *Paeonia suffruticosa*. Previous studies have extensively explored the clinical benefits of paeonol. However, comprehensive reviews on the cardiovascular protective effects of paeonol have not been conducted. The current review summarizes studies reporting on the protective effects of paeonol on the cardiovascular system. This study includes studies published in the last 10 years. The biological characteristics of *Paeonia suffruticosa*, pharmacological mechanisms of paeonol, and its toxicological and pharmacokinetic characteristics were explored. The findings of this study show that paeonol confers protection against atherosclerotic cardiovascular disease through various mechanisms, including inflammation, platelet aggregation, lipid metabolism, mitochondria damage, endoplasmic reticulum stress, autophagy, and non-coding RNA. Further studies should be conducted to elucidate the cardiovascular benefits of paeonol.

## Introduction

Atherosclerotic cardiovascular disease poses a significant health threat in many countries. Advances in medical technology have led to the discovery of drugs and interventional therapies for atherosclerotic cardiovascular disease (1). However, available drugs are associated with various adverse effects, including high tolerance (2, 3), rhabdomyolysis (4), and restenosis (5). It has been shown that natural compounds may confer protection against atherosclerotic cardiovascular disease through various mechanisms (6, 7).

Cortex Moutan, the root bark of *Paeonia suffruticosa*, has been widely used in traditional Chinese medicine to prevent diabetes (8, 9), arthritis (10), and cancer (11). Paeonol is a biologically active ingredient that is extracted from Cortex Moutan. In Asian countries, paeonol is commonly used in combination therapy for the management of the atherosclerotic cardiovascular disease (12). This review aims at elucidating the cardiovascular protective effects and pharmacological mechanisms of paeonol.

## Biological Characteristics of *Paeonia suffruticosa*

### Nomenclature of *Paeonia suffruticosa*

**Figure 1:**
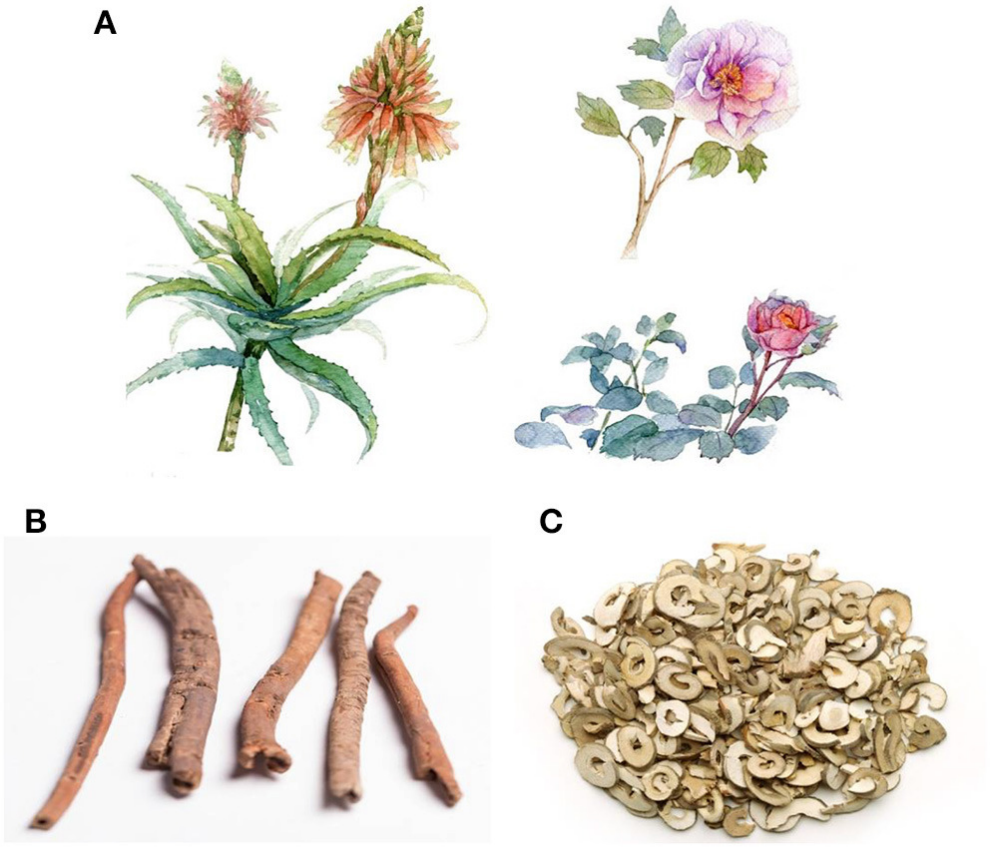
Whole plant and root bark of *Paeonia suffruticosa*. **(A)** *Paeonia suffruticosa* is a kind of plant with ornamental and medicinal values. **(B)** Cortex Moutan, the root bark of *Paeonia suffruticosa*, is a natural medicine with a variety of biological effects. **(C)** prepared slices of Cortex Moutan, containing various bioactive pharmacological compounds.

*Paeonia suffruticosa*, belonging to the Paeoniaceae family, is an ornamental plant that is widely distributed around the globe. Cortex Moutan, the root bark of *Paeonia suffruticosa*, has been used for nearly 2,000 years as a natural medicine for alleviating pathogenic heat from the blood, activation of blood circulation, and elimination of stasis (13, 14). Details on characteristics of *Paeonia suffruticosa* and Cortex Moutan are presented in Figure 1.

### Botanical Characteristics

Peonies predominantly grow in temperate Eurasia, northwest Africa, and western North America. However, wild peony is only found in China, where it was first domesticated (15). Perennial shrubs grow to 1.5 m in height with brown-gray stems. Its leaflets are long ovate or ovoids. Its flowers are solitary, single or double, with an irregular apex. The root system is well-developed, the root bark is yellow to brown, while the fleshy center is lignified (13, 16, 17). The bark is tubular or semi-tubular, with cracks along the longitudinal section. In addition, the bark is slightly curled inward, 3–8 cm long, 0.5–1.2 cm in diameter, and 0.1–0.4 cm thick. The outer skin is grayish-brown or reddish-brown. The cork drop is pink with protruding lenticels while the inner surface is light brown or gray-yellow with a fine longitudinal texture and shiny crystals. It has a special aroma and a slightly bitter taste (18).

### Paeonol: The Main Therapeutic Ingredient of *Paeonia suffruticosa*

**Figure 2:**
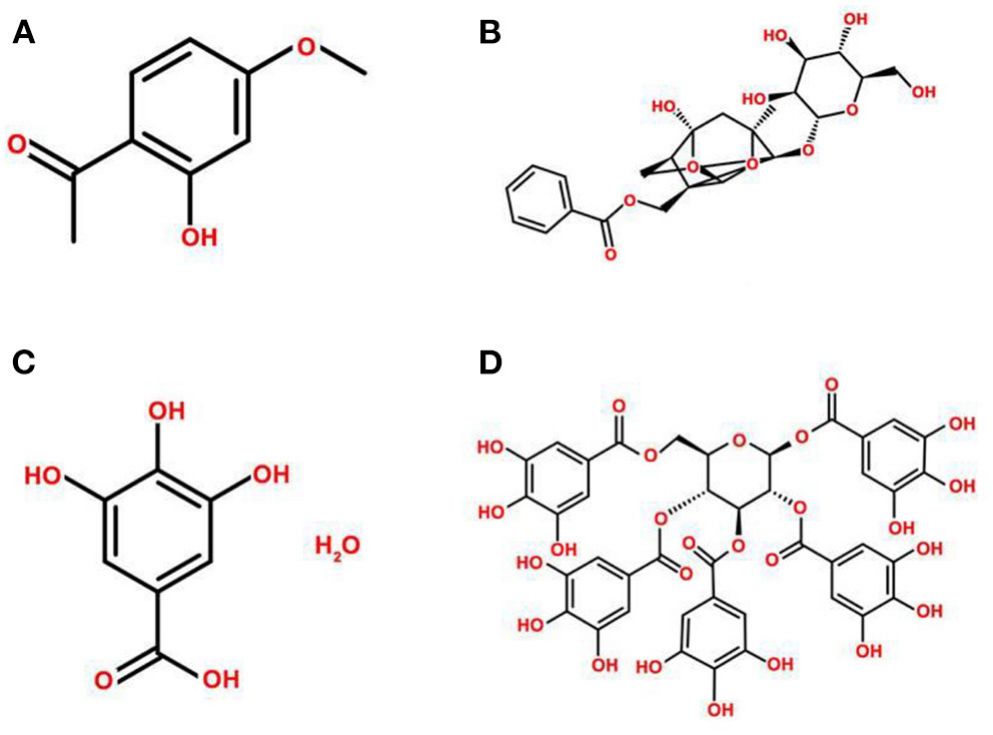
Chemical structures of the main active ingredients of *Paeonia suffruticosa*. **(A)** paeonol, **(B)** paeoniflorin, **(C)** gallic acid, **(D)** 1,2,3,4,6-pentakis-O-galloyl-β-D-glucose.

*Paeonia suffruticosa* contains various bioactive constituents, including monoterpene glycosides, flavonoids, gallic derivatives, and triterpenoids, especially phenols (19–22). The main active ingredients include paeonol, paeoniflorin, gallic acid, and 1,2,3,4,6-pentakis-O-galloyl-β-D-glucose, and the chemical structures are presented in Figure 2. The extraction methods of active ingredients include organic solvent extraction, ultrasonic-assisted extraction, steam distillation, and CO_2_ supercritical fluid extraction (23–25).

In recent years, paeonol has been widely used in medical health care and as a food supplement (26). Although it has several pharmacological benefits against various diseases, paeonol is mainly used for the treatment of atherosclerotic cardiovascular disease (27), such as myocardial ischemia, myocardial infarction, atherosclerotic stroke, and myocardial ischemia-reperfusion injury (28).

### Extraction Methods and Quality Control

The main extraction methods for paeonol include organic solvent extraction, steam distillation, and CO_2_ supercritical fluid extraction (23, 25). The steam distillation method is relatively simple and has a high transfer rate, while the CO_2_ supercritical fluid extraction method has the advantages of low temperatures and high efficiency, and is widely used for paeonol extraction. Artificial synthesis can also yield large amounts of paeonol at low costs for industrial production. Methods such as microwave-assisted extraction and ionic liquid extraction have also been widely used in recent years (29, 30).

Based on the provisions of the 2015 edition of Chinese pharmacopeia, the total ash content of the cortex of medicinal materials should not exceed 5.0% (General Rule 2302). Paeonol (C9H10O3) content, as determined by high-performance liquid chromatography (HPLC, General Rule 0512), should not be <1.2%. By determining alcohol-soluble extract (General Rule 2201) under the hot immersion method, using ethanol as a solvent, the extract shall not be <15.0% (31).

## Pharmacological Mechanisms of Paeonol on Atherosclerotic Cardiovascular Disease

### Inflammation

Inflammatory processes are involved in the pathogenesis of various cardiovascular diseases (32–34). Paeonol exhibits significant anti-inflammatory properties in colitis (35), arthritis (36), neuroinflammation (37), and other conditions. Similarly, paeonol can exert anti-inflammatory effects to inhibit the progression of atherosclerotic cardiovascular disease.

Cytokines are crucial mediators of inflammation. Studies have revealed that paeonol inhibits the formation and development of atherosclerosis by suppressing the release of inflammatory cytokines. In atherosclerosis rabbit models, paeonol intervention decreased atherosclerotic plaques and normalized serum levels of tumor necrosis factor (TNF)-α, interleukin (IL)-1β, and C-reaction protein (CRP) (38). Another study reported that paeonol inhibited lipopolysaccharide (LPS)-induced expression of nitrous oxide (NO), prostaglandin (PG) E2, and IL-6 (39). These findings confirm that paeonol inhibits the release of various inflammatory factors.

Inhibitory effects of paeonol on inflammatory factors are mainly associated with the regulation of various inflammatory pathways (40). Kim et al. reported that paeonol inhibited the migration of human umbilical vein endothelial cells (HUVECs) and the ability to form new blood vessels under the induction of basic fibroblast growth factor (bFGF). The study proved that paeonol inhibited Akt signaling pathways and the activity of matrix metalloproteinases (MMPs), hence suppressing angiogenesis and metastasis (41). Moreover, paeonol suppressed oxidized low-density lipoprotein (ox-LDL)-induced endothelial cell (EC) apoptosis by inhibiting the p38 mitogen-activated protein kinase (MAPK)-nuclear factor-kappa B (NF-κB) signaling pathway (42, 43). Choy et al. evaluated the effects of paeonol on LPS-induced inflammatory damage in HUVECs and C57BL/6J mice. It was found that paeonol suppressed LPS-induced EC dysfunction by independently inhibiting Toll-like receptor (TLR) 4 and bone morphogenetic protein (BMP) 4 signaling pathways (44). Other studies reported that the inhibitory effects of paeonol on inflammation might be related to the regulation of microRNA (miRNA) expression (45, 46). In dog models, paeonol significantly reduced the area of myocardial infarction and the release of myocardial enzymes (47). Similarly, paeonol conferred protection against myocardial infarction in a rat model (48, 49). Zhou et al. used a coronary artery left ventricular branch ligation model to explore the effect of paeonol on ventricular remodeling in rabbits with myocardial infarction. Paeonol treatment improved ventricular function and significantly suppressed TLR4 and TNF-α mRNA expression levels. Paeonol down-regulated the expression of downstream inflammatory factor, TNF-α, by inhibiting the TLR4 signaling pathway to improve ventricular remodeling after myocardial infarction (50). Paeonol was also found to reverse ventricular remodeling after myocardial infarction by inhibiting NF-κB-p65 and down-regulating MMP-9 (51). Studies also suggested that paeonol regulated ventricular remodeling through the transforming growth factor (TGF) -β signaling pathway (52–54). Therefore, inhibitory effects of paeonol on inflammatory factors are mainly associated with the regulation of MAPK, TLR, MMP, and TGF pathways, and may be correlated with the regulation of apoptosis, autophagy, and the transcription of miRNAs.

Monocyte adhesion, induced by the adhesion factors, is an essential step in the pathogenesis of atherosclerosis. Studies have confirmed the negative role of adhesion factors, especially vascular cell adhesion molecule (VCAM)-1 and intercellular adhesion molecule (ICAM)-1 on the development of cardiovascular diseases (55, 56). Levels of these factors may serve as markers of cardiovascular events (57). Nizamutdinova et al. reported that paeonol inhibited the production of ICAM-1. The binding of monocytes to ECs was inhibited by paeonol, probably due to the inhibition of p38, extracellular signal-regulated kinase (ERK), and NF-κB signaling pathways. Inhibition of these signaling pathways eventually altered the regulatory effects of TNF-α on ICAM-1 (58). Similarly, paeonol was shown to inhibit VCAM-1 expression through the p38 and ERK1/2 signal transduction pathways in rat aortic ECs (RAECs) (59, 60). In vascular ECs (VECs) extracted from the thoracic aorta of ox-LDL-induced injury rats, paeonol conferred endothelium protective effects by inhibiting monocyte adhesion to VECs and blocked the activation of phosphoinositide 3-kinase (PI3K)/Akt/NF-κB signaling pathway (61). In summary, paeonol regulates the expression of VCAM-1, ICAM-1 and inhibits the adhesion of monocytes by regulating NF-κB, ERK, and other inflammatory pathways.

Paeonol prevents the development of atherosclerosis by inhibiting the proliferation of vascular smooth muscle cells (VSMCs) (62, 63). A study reported that paeonol significantly inhibited the expression of proliferating cell nuclear antigen and the proliferation of VSMCs (64). Wu et al. found that paeonol down-regulated the expression of potent vasoconstrictor peptide endothelin (ET)-1, and inhibited the proliferation of VSMCs by suppressing the diacylglycerol-protein kinase C signaling pathway (65). Paeonol suppressed the release of vascular endothelial growth factor (VEGF) and platelet-derived growth factor B in a co-culture system of VSMCs and VECs. In addition, paeonol inhibited the proliferation of VSMCs through the Ras-Raf-ERK1/2 signaling pathway (66). Recent studies revealed that paeonol inhibited the invasion and proliferation of VSMCs. This process was accompanied by increased expression of microtubule-associated protein light chain 3 (LC3), degradation of p62, and the appearance of autophagosomes in the arterial middle layer. It was postulated that paeonol up-regulated autophagy and activated adenosine monophosphate-activated protein kinase (AMPK)/mechanistic target of rapamycin (mTOR) signaling pathway to inhibit the proliferation of VSMCs (67). Therefore, paeonol inhibits the proliferation of VSMCs by regulating diacylglycerol-protein kinase C, ERK signaling pathways, and autophagy. Details of experiments for the anti-inflammatory mechanisms of paeonol are presented in Table 1.

**Table 1:** Experiments on the anti-inflammatory mechanism of paeonol in atherosclerotic cardiovascular disease.

**Authors**	**Experimental model**	**Experimental method**	**Signaling molecules involved (paeonol group)**	**References**
Li et al.	High-fat-diet-induced atherosclerotic rabbit model	TBARS, Radioimmunity, ELISA, Immunohistochemical	TNF-α↓, IL-1β↓, CRP↓, NF-κB-p65↓	(38)
Chae et al.	LPS-induced RAW 264.7 cells	MTT, ELISA, Western blot, qRT-PCR	PGE2↓, IL-6↓, COX-2↓, ERK↓	(39)
Kim et al.	bFGF-induced HUVECs	Matrigel plug assay, Western blot, Gelatin zymographic assay	Akt↓, MMP-9↓, MMP-2↓	(41)
Bao et al.	Ox-LDL-induced HUVECs	Flow cytometry, DCFH-DA, RT-PCR, Western blot, Immunofluorescence	LOX-1↓, ROS↓, Bcl-2↑,p38/MAPK↓, NF-κB↓, caspase-3↓	(43)
Choy et al.	LPS-induced HUVECs	Western blot, Flow cytometry, Wire electromyography	TLR4↓, BMP-4↓, ROS↓, MAPK↓, iNOS↓, caspase-3↓	(44)
Liu et al.	Apolipoprotein E-knockout mice atherosclerosis model	ELISA, Western blot, qRT-PCR, Electron microscopy, Laser microscopy, Double luciferase gene report test	miRNA-223↑, IL-β1↓, IL-6↓,ICAM-1↓, VCAM-1↓, STAT3↓, p-STAT3↓	(45)
Liu et al.	Ox-LDL-induced VECs	qRT-PCR, Western blot, ELISA	miRNA-21↓, TNF-α↓, PTEN↑	(46)
Zhou et al.	LAD ligation induced acute myocardial rat model	Hematoxylin-eosin staining, Western blot	NF-κB-p65↓, MMP-9↓	(51)
Shi et al.	LAD ligation induced acute myocardial rat model	Hematoxylin-eosin staining, RT-PCR, Western blot	TGF-β1↓, Smad 2↓	(52)
Shi et al.	LAD ligation induced acute myocardial rat model	qRT-PCR, Western blot	Smad 7↑, Smad 2↓, Smad 3↓	(53)
Galkina et al.	TNF-α-induced HUVECs	Western blot, Immunofluorescence, Adhesion assay, ELISA	ICAM-1↓, NF-κB/p65↓, p38↓, ERK↓	(56)
Pan et al.	TNF-α-induced RAECs	Adhesion assay, ELISA, Western blot	VCAM-1↓, p38↓, ERK 1/2↓	(59)
Wang et al.	Ox-LDL-induced VECs	Adhesion assay, Immunofluorescence, Western blot	VCAM-1↓, MAPKs↓, JNK1/2↓, ERK1/2↓, p38↓	(60)
Yuan et al.	Ox-LDL-induced VECs	MTT, qPCR, Western blot, Adhesion assay	miRNA−126↑, VCAM-1↓, PI3K↓, Akt↓, NF-κB↓	(61)
Zhang et al.	Rabbit vein graft model	Immunohistochemistry, Western blot, TUNEL assay	PCNA↓, VCAM-1↓	(63)
Meng et al.	TNF-α-induced VSMCs	Immunofluorescence, Transwell assay, ELISA, Western blot	IL-1β↓, IL-6↓, caspase-3↓, caspase-9↓, Bax↑, Bcl-2↓	(64)
Chen et al.	Coculture model of VSMCs and VECs	Immunofluorescence, Western blot	VEGF↓, Ras↓, pRaf↓, pERK↓	(66)
Wu et al.	Ox-LDL-induced VSMCs	Immunofluorescence, BrdU assay, Flow cytometry, TEM, Western blot	LC3II↑, p62↓, pAMPK↑, mTOR↓	(67)

*DCFH-DA, 2′,7′-dichlorofluorescein diacetate; bFGF, basic fibroblast growth factor; Bax, Bcl-2 associated protein X; BMP, bone morphogenetic protein; JNK, c-Jun N-terminal kinase; CRP, C-reaction protein; COX, cyclooxygenase; ELISA, enzyme-linked immunosorbent assay; ERK, extracellular signal-regulated kinase; HUVEC, human umbilical vein endothelial cell; iNOS, inducible nitric oxide synthase; ICAM, intercellular adhesion molecule; IL, interleukin; LOX, lectin-like oxidized low-density lipoprotein receptor; LAD, left anterior descending branch; LPS, lipopolysaccharide; MMP, matrix metalloproteinase; mTOR, mechanistic target of rapamycin; MAPK, mitogen-activated protein kinase; NF-κB, nuclear factor-kappa B; ox-LDL, oxidized low density lipoprotein; PTEN, phosphatase and tensin homolog; PCNA, proliferating cell nuclear antigen; PG, prostaglandin; LC3, protein light chain 3; RAEC, rat aortic endothelial cell; ROS, reactive oxygen species; RT-PCR, reverse transcription-polymerase chain reaction; STAT, signal transducer and activator of transcription; TUNEL, terminal deoxynucleotidyl transferase-mediated dUTP nick end labeling; MTT, thiazolyl blue tetrazolium bromide; TBARS, thiobarbituric acid reactive substance; TLR, Toll-like receptor; TGF, transforming growth factor; TEM, transmission electron microscopy; TNF, tumor necrosis factor; VCAM, vascular cell adhesion molecule; VEC, vascular endothelial cell; VEGF, vascular endothelial growth factor; VSMC, vascular smooth muscle cell. ↑ represents up-regulation of expression, and ↓ represents down-regulation of expression*.

### Abnormal Lipid Metabolism

Abnormal lipid metabolism is one of the independent risk factors for atherosclerosis (68). Paeonol suppressed serum levels of total cholesterol, triglyceride, low-density lipoprotein, very low-density lipoprotein, apolipoprotein B100, as well as total cholesterol levels in the aorta and liver (69). Moreover, paeonol increased high-density lipoprotein levels, improved blood and plasma viscosity in rabbit models (70). A study reported that paeonol could enhance the activity of hepatic lipase, lipoprotein lipase and inhibit the activity of 3-hydroxy-3-methyl glutaryl coenzyme A reductase (HMGCR) (71).

**Figure 3:**
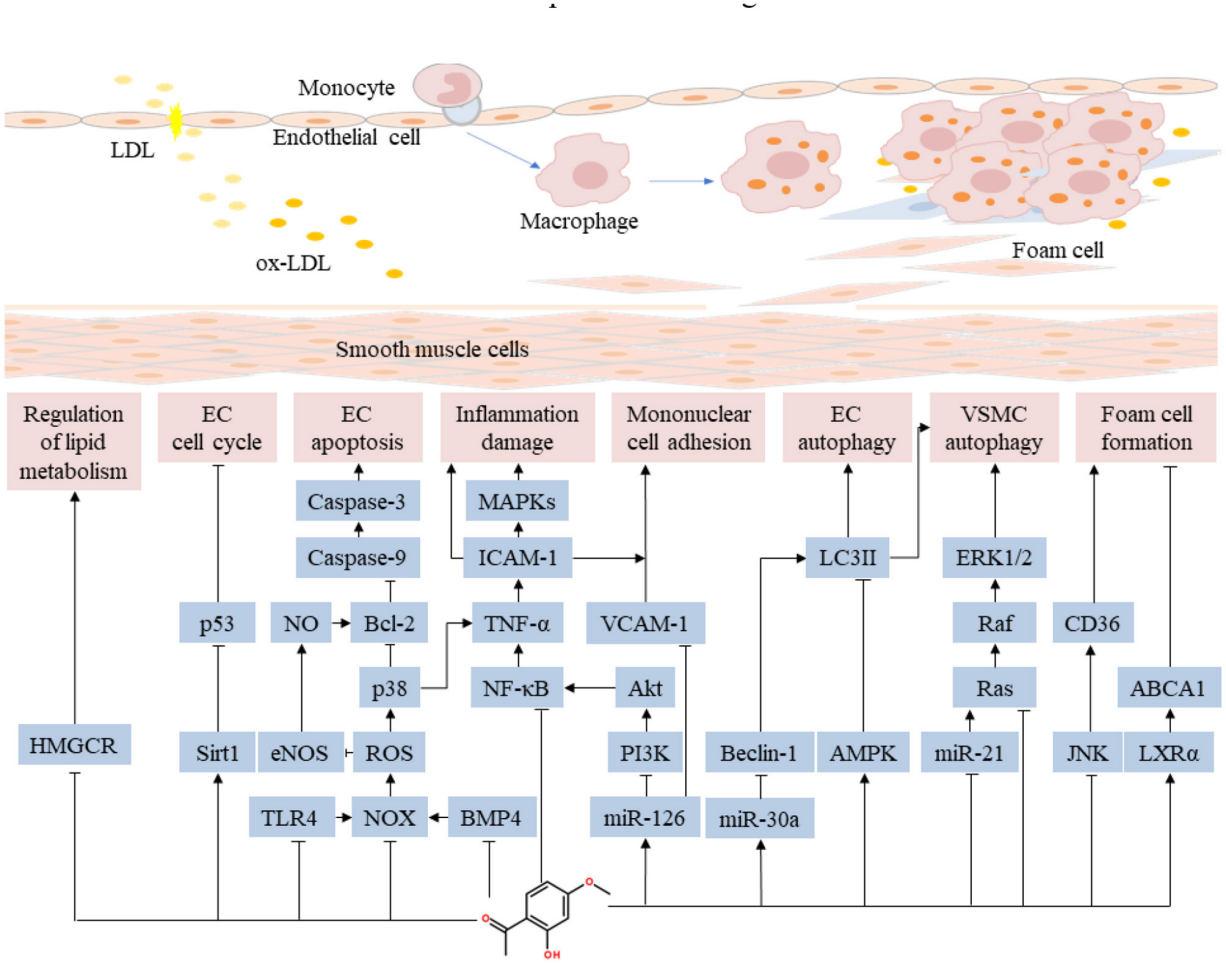
Pharmacological mechanisms of paeonol on anti-inflammatory and regulating lipid metabolism in atherosclerotic cardiovascular disease. Paeonol confers anti-inflammatory effect by suppressing the release of inflammatory cytokines through a variety of pathways, inhibiting the monocyte adhesion and the proliferation of VSMCs. Paeonol regulates lipid metabolism by inhibiting lipid synthesis, enhancing the activity of lipase, and regulating the reverse transport process in macrophages. ABCA1, adenosine triphosphate-binding cassette transporter A1; JNK, c-Jun N-terminal kinase; EC, endothelial cell; eNOS, endothelial nitric oxide synthase; ERK, extracellular signal-regulated kinase; ICAM, intercellular adhesion molecule; LXRα, liver X receptor-alpha; MAPK, mitogen-activated protein kinase; NOX, nitrogen oxide; NO, nitrous oxide; NF-κB, nuclear factor-kappa B; PI3K, phosphoinositide 3-kinase; ROS, reactive oxygen species; HMGCR, recombinant 3-hydroxy-3-methylglutaryl coenzyme A reductase; TLR, Toll-like receptor; TNF, tumor necrosis factor; VCAM, vascular cell adhesion molecule; VSMC, vascular smooth muscle cell.

Foam cells, typical pathologic cells in atherosclerotic plaques, are formed by macrophage phagocytosis of ox-LDL and intracellular lipids (72–74). Reverse cholesterol transport is an important mechanism for inhibiting the formation of foam cells (75, 76). A study found that paeonol inhibited cholesterol accumulation in macrophages following treatment with ox-LDL. Paeonol significantly elevated mRNA and protein expression of adenosine triphosphate (ATP)-binding cassette transporter A1 (ABCA1). Notably, ABCA1 inhibitors abrogated the effects of paeonol on cholesterol efflux and accumulation. This result suggested that paeonol conferred protection to macrophages by promoting cholesterol efflux (77). In RAW264.7 macrophages and apolipoprotein E-knockout mice, paeonol reduced lipid accumulation in macrophages by suppressing ox-LDL uptake and by enhancing cholesterol outflow. A small hairpin RNA targeting heme oxygenase (HO)-1 inhibited paeonol-induced benefits on the expression of c-Jun, CD36, ABCA1, calpain activity in macrophages (78). Collectively, these results show that paeonol regulates lipid metabolism by inhibiting lipid synthesis, enhancing the activity of lipase, and regulating the reverse transport process in macrophages. Pharmacological mechanisms of paeonol on anti-inflammatory and regulating lipid metabolism in atherosclerotic cardiovascular disease are presented in Figure 3.

### Platelet Aggregation

**Figure 4:**
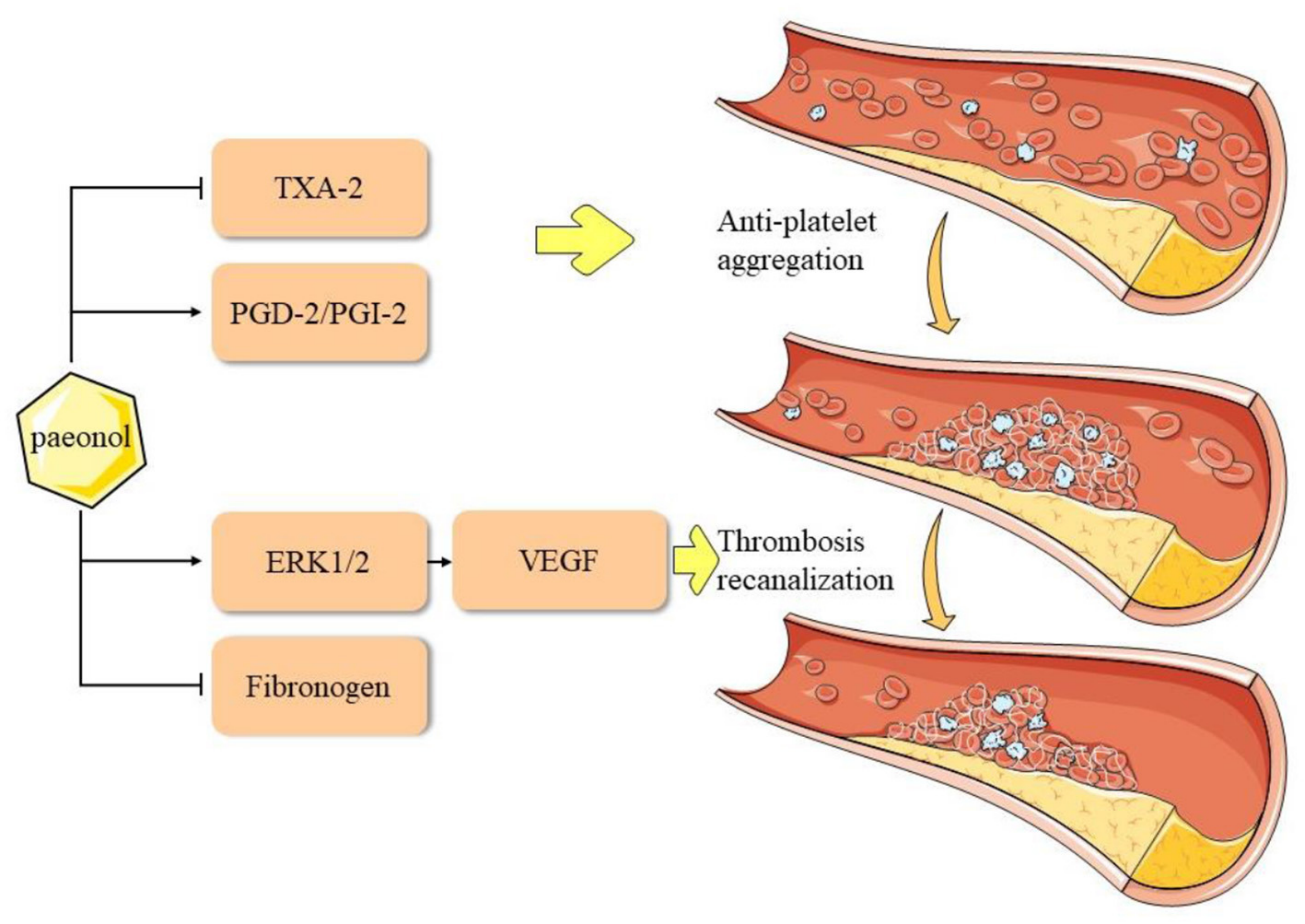
Pharmacological mechanisms of paeonol in inhibition of platelet aggregation in atherosclerotic cardiovascular disease. ERK, extracellular signal-regulated kinase; PG, prostaglandin; PGI2, prostacyclin; TX, thromboxane; VEGF, vascular endothelial growth factor.

Adhesive aggregation of platelets and the release of EC growth factors can stimulate smooth muscle cells (SMCs) to migrate into the intima (79–81), thereby accelerating the formation of foam cells. Moreover, in the unstable plaque rupture process, high platelet aggregation causes fatal thrombosis (82), which leads to myocardial infarction. Previous studies reported that paeonol reduced whole blood viscosity, plasma viscosity, and platelet adhesion, which exerted a positive effect on hemodynamics (83, 84). Doble et al. performed a platelet aggregation test using paeonol analogs and built a back-propagation neural network model for testing. It was found that paeonol analogs could not interact with the cyclooxygenase (COX)-1 enzyme to inhibit platelet activity, which suggested that the effect of paeonol on platelet aggregation may involve other mechanisms (85). Koo et al. reported that paeonol could promote blood circulation by inhibiting platelet aggregation and coagulation (86). Another study reported that paeonol analogs effectively and selectively inhibited the arachidonic acid-induced aggregation of rabbit platelets. Furthermore, these analogs inhibited the arachidonic acid-induced formation of thromboxane (TX) A2 and promoted the production of PGD2 (87). Ye et al. elucidated that D-dimer and TXB2 levels were suppressed and the expression levels of ERK 1/2 and VEGF were significantly increased after paeonol treatment in a rat model of thrombus recanalization (88). In summary, paeonol inhibits platelet aggregation through the regulation of TX, PG, and enhances thrombus recanalization *via* the ERK1/2 signaling pathway (Figure 4).

### Mitochondria Damage and Apoptosis

Oxidative stress plays a significant role in the development of atherosclerosis (89–91). Studies have shown that paeonol confers antioxidant properties in various cardiovascular diseases (92, 93). Paeonol was shown to enhance NO production while reducing reactive oxygen species (ROS) production (94), thereby preventing EC damage caused by ROS.

Mitochondria are the main sources of intracellular ROS and are closely associated with oxidative stress. Atherosclerosis, inflammation (95), hyperglycemia (96), hyperlipidemia (97), and other risk factors can lead to mitochondrial damage and stimulate the overproduction of ROS. This process promotes the apoptosis of ECs and SMCs, resulting in atherosclerosis (98). A previous study reported that paeonol inhibited hypoxia-induced damage of primary human pulmonary artery SMCs (PASMCs) mitochondrial damage *in vitro*. Moreover, paeonol improved adverse effects of decreased ATP production, morphological changes, mitochondrial polarization, and increased ROS generation. In addition, paeonol caused significant changes in mitochondrial-dependent apoptosis through peroxisome proliferator-activated receptor (PPAR) γ coactivator (PGC)-1α (99). These results show the protective effects of paeonol on mitochondrial under hypoxic conditions and confirm the crucial role of PGC-1α in PASMCs apoptosis.

Studies have shown that paeonol plays a protective role in the myocardium by regulating cell apoptosis. Li et al. reported the protective effect of paeonol on myocardium in myocardial infarction in rats. The effect was correlated with the antioxidant defense system through the activation of the nuclear factor erythroid 2-related factor (Nrf2) signaling pathway and the regulation of Bcl-2 associated protein X (Bax), Bcl-2, and caspase-3 (51). Other findings suggested that the protective effect of paeonol on myocardium in ischemia-reperfusion injury was associated with inhibition of cell apoptosis (100). The hypoxic/reoxygenated rat cardiomyocyte model showed that paeonol could down-regulate the expression of apoptosis-related proteins to restore the viability of H9c2 cells. The mechanism was correlated with the activation of the PI3K/Akt pathway (101). In conclusion, paeonol exerts cardiovascular protective effect by protecting mitochondria and inhibiting apoptosis, and the mechanism is related to the activation of PGC-1α, Nrf2, and PI3K pathways.

### Endoplasmic Reticulum Stress

Pathological stimuli including hypoxia, ischemia, inflammation, and oxidative stress interrupt the homeostatic function of the endoplasmic reticulum, leading to the accumulation of unfolded proteins, a condition referred to as ERS (102). Endoplasmic reticulum stress determines cell fate and function (103, 104). A large number of studies show that ERS aggravates cardiovascular diseases (105, 106), especially atherosclerosis (107, 108).

Studies have shown that paeonol plays a cardiovascular protective role by inhibiting ERS. Choy et al. investigated the protective mechanism of paeonol against tunicamycin-induced ERS in isolated mouse aortas ECs and HUVECs. The results confirmed that paeonol treatment increased ERS markers, oxidative stress, and reduction of NO bioavailability induced by tunicamycin were reversed. Paeonol increased the expression of AMPK, PPARδ while restoring the decreased phosphorylation of endothelial nitric oxide synthase. The study showed that paeonol alleviated tunicamycin-induced vascular endothelial dysfunction by inhibition of ERS and oxidative stress, thus elevating NO bioavailability *via* the AMPK/PPARδ signaling pathway (109). Similar studies explored the vascular protective effects of paeonol on ER stress-induced endothelial dysfunction in mice (110). The findings indicated that paeonol preserved endothelial function in mice induced by tunicamycin *via* inhibiting ERS.

### Autophagy

Autophagy is a highly conserved mechanism of lysosome-mediated protein and organelle degradation that plays a crucial role in maintaining cellular homeostasis (111). In the cardiovascular system, autophagy appears to be essential to heart and vessel homeostasis and function. However, defective or excessive autophagy activity is associated with atherosclerosis (112, 113).

Wu et al. reported that paeonol restricted the development of atherosclerosis in apolipoprotein E-knockout mice and decreased the amount of VSMCs in the media layer. Paeonol enhanced the formation of autophagosomes and up-regulated the expression of LC3II. In addition, Paeonol induced phosphorylation of AMPK and reduced phosphorylation of mTOR. These results showed that paeonol inhibited the proliferation of VSMCs by up-regulating autophagy and activating the AMPK/mTOR signaling pathway (69). Similarly, recent research found that paeonol prevented lipid metabolism dysfunction in palmitic acid-induced HepG2 injury by promoting Sirtuin 1-FoxO1 autophagy-related pathway (114).

Li et al. used ox-LDL-induced rat VECs as a model system to elucidate the protective effect of paeonol on VECs injury. The study showed that paeonol significantly reduced ox-LDL-induced formation of autophagy vacuoles and the expression of LC3II in VECs. Moreover, the study reported that ox-LDL decreased miRNA-30a and increased Beclin-1 expression, whereas pretreatment with paeonol reversed the process of regulation (115). Another research investigated the molecular mechanisms of the crosstalk between apoptosis and autophagy subjected to myocardial ischemia/reperfusion (I/R) injury. The result showed that paeonol significantly improved cardiac function after I/R. Compared with vehicle treatment, paeonol significantly downregulated the cleaved forms of caspase-8, caspase-9, caspase-3 protein expression, and myocardial I/R-induced autophagy was significantly reversed by paeonol treatment (116). Similar research showed that paeonol could induce VSMCs autophagy by activating the class III PI3K/Beclin-1 signaling pathway, thus ultimately inhibiting VSMCs apoptosis (117).

In conclusion, paeonol exerts cardiovascular protective effects by regulating autophagy, and the mechanism may be related to AMPK and Sirtuin 1 pathways. Recent studies have found that the regulatory effect of paeonol on autophagy may be related to miRNA and the crosstalk between autophagy and apoptosis, which may be the focus of further research on the mechanism of the cardiovascular protective effect of paeonol.

### Non-coding RNA

Non-coding RNA is a class of transcripts that do not encode proteins and include miRNA, long non-coding RNA, and circular RNA (118). Studies have found that miRNAs are closely associated with cardiovascular disease. The potential of miRNAs as therapeutic targets for heart and vascular disease, and the use of miRNAs as novel biomarkers (119, 120).

Shi et al. used high-fat-diet-induced hyperlipidemic rats as a model to investigate if paeonol could inhibit nucleotide-binding oligomerization domain (NOD)-like receptor family pyrin domain containing 3 (NLRP3) inflammasome by elevating plasma-derived exosomal miRNA-223. *In vivo* experiments confirmed that paeonol increased the survival rate of RAECs and the expression of exosomal miRNA-223. Moreover, paeonol decreased the expression of NLRP3, caspase-1, and ICAM-1. These results showed that paeonol inhibited the downstream NLRP3 inflammasome pathway by increasing the level of miRNA-223 in plasma-derived exosomes of hyperlipidemic rats (121). Yuan et al. explored the effects of paeonol on miRNA-126 expression and its ability to inhibit monocyte adhesion to ox-LDL-injured VECs. Results showed that paeonol promoted miRNA-126 expression and suppressed VCAM-1 expression at the mRNA and protein level. In addition, it inhibited monocyte adhesion to ox-LDL-injured VECs by upregulating miRNA-126 expression. Furthermore, it was demonstrated that paeonol blocked the activation of the NF-κB signaling pathway by promoting miRNA-126 expression (63). Another study reported that paeonol alleviated ox-LDL-induced VECs injury by targeting miRNA-30a thus inhibiting excessive autophagy (115).

In summary, miRNAs such as miRNA-223, miRNA-126, and miRNA-126 play important roles in the protective activity of paeonol on cardiovascular disease. Notably, miRNAs in exosomes can be targeted for the treatment of atherosclerotic cardiovascular disease.

### Intestinal Flora

Alterations in the composition and function of intestinal flora, known as gut microflora dysbiosis, can accelerate the progression of a variety of diseases (122). The interaction between intestinal flora and natural medicine is crucial for host health (123).

**Figure 5:**
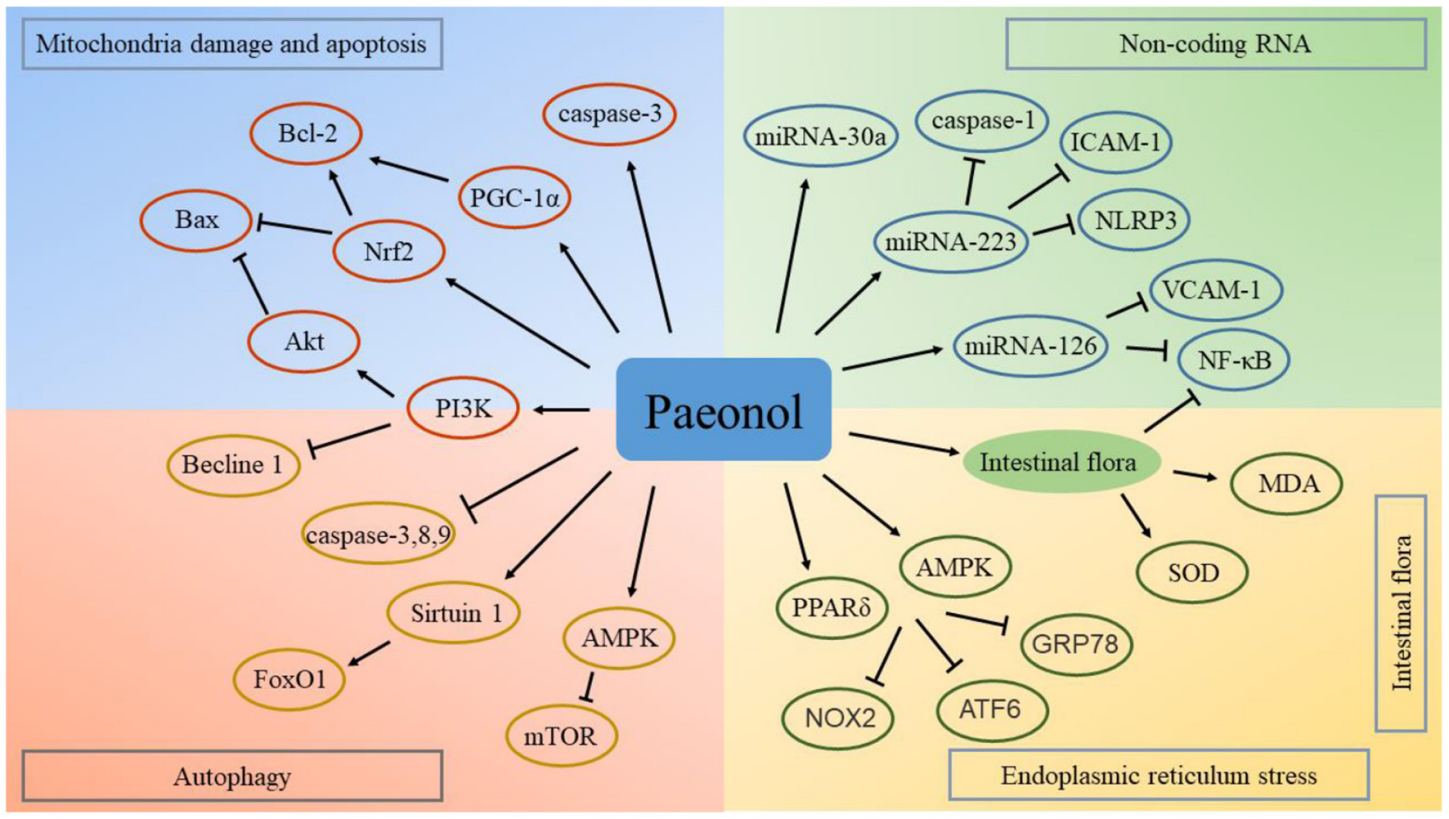
Potential targets and mechanisms of paeonol in the treatment of atherosclerotic cardiovascular disease. ATF6, activating transcription factor 6; AMPK, adenosine monophosphate-activated protein kinase; Bax, Bcl-2 associated protein X; GRP78, glucose-regulated protein 78; ICAM, intercellular adhesion molecule; MDA, malonaldehyde; mTOR, mechanistic target of rapamycin; NOX2, nicotinamide adenine dinucleotide phosphate oxidase; Nrf2, nuclear factor erythroid 2-related factor; NF-κB, nuclear factor-kappa B; NLRP3, nucleotide binding oligomerization domain-like receptor family pyrin domain containing 3; PPAR, peroxisome proliferator-activated receptor; PGC, peroxisome proliferator-activated receptor γ coactivator; PI3K, phosphoinositide 3-kinase; SOD, superoxide dismutase; VCAM, vascular cell adhesion molecule.

Previous studies have confirmed that natural medicines can be used for the treatment of a variety of intestinal and digestive system diseases by regulating intestinal flora (124, 125). Intestinal flora plays an important role in the treatment of cardiovascular diseases using natural compounds (126). Studies have explored the regulatory effect of paeonol on the intestinal flora. Paeonol exerts anti-inflammatory effects by regulating intestinal flora (127). In addition, paeonol regulates the brain-gut axis mediated NF-κB signaling pathway and exerts a therapeutic effect in cerebral infarction (128). The cross-talk between gut microbiota and the heart presents a new therapeutic target for paeonol in the treatment of cardiovascular diseases. Potential targets and mechanisms of paeonol in the treatment of atherosclerotic cardiovascular disease are presented in Figure 5.

## Toxicology and Pharmacokinetics of Paeonol

Studies have not evaluated the toxicity and safety of paeonol, however, its pharmacokinetics have been reported. Oral paeonol is well-absorbed in the entire intestines, except for the colon. Paeonol is absorbed through passive diffusion (129), which conforms to the first-order elimination kinetics (130), and the drug-time curve has double peaks (131).

The ultra-HPLC-tandem mass spectrometry method was used to assess the absorption of orally administered paeonol in rats. The findings showed that paeonol was distributed in various tissues without long-term accumulation. Paeonol and its metabolites were mainly distributed in the kidney, liver, and heart. Paeonol could pass through the blood-brain barrier, however, its level in the brain dropped rapidly after 10 min (132). This conclusion was inconsistent with previous reports using gas chromatography-mass spectrometry showing that paeonol could not easily penetrate the blood-brain barrier but accumulated in the brain for long periods (133). Reverse-phase HPLC revealed that paeonol was mainly excreted in urine (134, 135). Paeonol was affected by the interactions of coexisting ingredients during preparation (136). For example, co-administration could alter pharmacokinetics and tissue distribution of paeonol in rats, mainly in the heart and brain (137).

Wu et al. used HPLC to measure paeonol concentration in human plasma after oral medication and found that the linear relationship was acceptable within the concentration range of 10–500 ng/ml. Analysis showed no statistical differences in the main pharmacokinetic parameters of the tablet and capsule dosage forms of the drug (138). Other studies reported that the pharmacokinetics of paeonol varied with administration routes (139). The absolute bioavailability of paeonol after intramuscular injection was 68.68% (140), while the absolute bioavailability of orally administered paeonol was 28.92%, which was relatively low (141). Poor water solubility and stability as well as high volatility at room temperatures limited clinical applications of paeonol (28).

Several studies improved the absorption efficiency and bioavailability of paeonol by changing the administrative route (142, 143). Paeonol is administered in conventional formulations such as tablets, topical gels, hydrogels, and polymer delivery systems such as nanocapsules and polymeric nanoparticles, microemulsions, liposomes, and lipid-based nanoparticles (144–146). Studies on dosage forms focused on transdermal delivery. Isopropyl myristate, cremophor, and polyethylene-glycol were used to prepare microemulsion gels, and glycerol monooleate to prepare cubic gels. The two formulations had higher skin permeabilities compared with paeonol solution, and their relative bioavailability was higher by 1.28 times and 1.51 times, respectively, compared with paeonol solution (147). Using skin-blood synchronous microdialysis coupled with the liquid chromatograph-mass spectrometer, Liu et al. reported that stable concentrations of paeonol microemulsion gel in blood were higher than that of general dosage forms (148, 149). Shi et al. used the Franz diffusion cell and reported that the positive ion liposome gel had superior stabilities and permeabilities compared with conventional gels (150). Ethanol injection was used to prepare paeonol-loaded nanovesicles, while HPLC was used to analyze the concentrations of paeonol in rat plasma after transdermal administration. The nanovesicle formulation showed an enhanced transdermal flux, and paeonol bioavailability was significantly improved in line with the one-compartment absorption model. Similarly, nanocapsules improved the bioavailability and pharmacological properties of paeonol (151, 152). These new drug delivery systems have improved the stability, bioavailability, and pharmacological properties of paeonol.

## Concluding Remarks

The cardiovascular effects of paeonol have been extensively studied. Paeonol suppresses inflammatory factor production by modulating signaling pathways such as MAPK, TLR, and ERK. In addition, paeonol improves lipid metabolism by inducing lipase activity and inhibiting lipid synthesis. Paeonol plays an important role in reverse cholesterol transport thus preventing the formation of foam cells. Furthermore, it protects mitochondria and reduces ROS accumulation. This is achieved by up-regulating cell autophagy and induction of cell apoptosis. Paeonol protects ischemic tissues and prevents platelet aggregation. Moreover, paeonol regulates ERS, non-coding RNA, and intestinal flora. Although the pharmacokinetics of paeonol has been extensively studied, its toxicological effects and safety have not been evaluated. High-quality clinical controlled trials should be conducted to explore the clinical benefits of paeonol in atherosclerotic cardiovascular diseases.

## Author Contributions

MW and LL: the conception and design of the study. ZY: the conception and design of the study and drafting the article. XL: drafting the article. XZ, SW and LL: revising the article critically. SY and LH: revising the article. All authors contributed to the article and approved the submitted version.

## Conflict of Interest

The authors declare that the research was conducted in the absence of any commercial or financial relationships that could be construed as a potential conflict of interest.

## Abbreviations

HMGCR: 3-hydroxy-3-methyl glutaryl coenzyme A reductase
AMPK: adenosine monophosphate-activated protein kinase
ATP: adenosine triphosphate
ABCA1: adenosine triphosphate-binding cassette transporter A1
bFGF: basic fibroblast growth factor
Bax: Bcl-2 associated protein X
BMP: bone morphogenetic protein
CRP: C-reaction protein
COX: cyclooxygenase
ERS: endoplasmic reticulum stress
EC: endothelial cell
ET: endothelin
ERK: extracellular signal-regulated kinase
HO: heme oxygenase
HPLC: high-performance liquid chromatography
HUVEC: human umbilical vein endothelial cell
ICAM: intercellular adhesion molecule
IL: interleukin
I/R: ischemia/reperfusion
LPS: lipopolysaccharide
MMP: matrix metalloproteinase
mTOR: mechanistic target of rapamycin
miRNA: microRNA
MAPK: mitogen-activated protein kinase
NO: nitrous oxide
NLRP3: NOD-like receptor family pyrin domain containing 3
Nrf2: nuclear factor erythroid 2-related factor
NF-κB: nuclear factor-kappa B
NOD: nucleotide binding oligomerization domain
ox-LDL: oxidized low-density lipoprotein
PPAR: peroxisome proliferator-activated receptor
PGC: peroxisome proliferator-activated receptor γ coactivator
PI3K: phosphoinositide 3-kinase
PG: prostaglandin
LC3: protein light chain 3
PASMC: pulmonary artery smooth muscle cell
RAEC: rat aortic endothelial cell
ROS: reactive oxygen species
SMC: smooth muscle cell
TX: thromboxane
TLR: Toll-like receptor
TGF: transforming growth factor
TNF: tumor necrosis factor
VCAM: vascular cell adhesion molecule
VEC: vascular endothelial cell
VEGF: vascular endothelial growth factor
VSMC: vascular smooth muscle cell.
